# Patient-ventilator asynchrony knowledge, attitudes, and practices among ICU health care professionals: questionnaire development

**DOI:** 10.36416/1806-3756/e20260010

**Published:** 2026-08-13

**Authors:** Mayson L A Sousa, Fabia Diniz-Silva, Sandra R Wilson, Juliana C Ferreira

**Affiliations:** 1. Department of Respiratory Therapy, College of Rehabilitation Sciences, Rady Faculty of Health Sciences, University of Manitoba, Winnipeg (MB) Canada.; 2. Divisao de Pneumologia, Instituto do Coracao, Hospital das Clinicas, Faculdade de Medicina, Universidade de Sao Paulo, Sao Paulo (SP) Brasil.; 3. Department of Medicine, Stanford University School of Medicine, Palo Alto (CA) United States.

**Keywords:** Respiration, artificial, Positive-pressure respiration, Respiratory insufficiency, Interactive ventilatory support, Critical care, Education, medical

## Abstract

**Objective:**

To describe the development and validation process of a questionnaire to assess the knowledge, attitudes, and practices of ICU health care professionals regarding patient-ventilator asynchrony.

**Methods:**

This was a knowledge, attitudes, and practices questionnaire development study. Item generation was guided by a literature-based conceptual framework, followed by expert review to establish face and content validity. The instrument was refined through specialists in mechanical ventilation and questionnaire design. The tenth version was piloted in Portuguese among ICU health care professionals, including a 30-day test-retest assessment.

**Results:**

The final version of the questionnaire comprised 24 items (knowledge, 14 items; attitudes, 4; and practices, 6). Of the 83 ICU health care professionals who consented to participate, 49 completed at least 80% of the questionnaire twice and were included in the analysis. The knowledge section showed acceptable internal consistency (Cronbach’s alpha = 0.73) and moderate test-retest reliability (intraclass correlation coefficient = 0.48; 95% CI, 0.24-0.67). Median knowledge scores were 71 [57-79] at test and 64 [50-79] at retest, out of 100 (p = 0.052). Attitude scores also showed good test-retest reliability (intraclass correlation coefficient = 0.68; 95% CI, 0.50-0.81) and a moderate correlation (r = 0.60; p < 0.001), with median scores of 75 (out of 100) in both rounds. Practice item responses were generally consistent between test and retest.

**Conclusions:**

We developed a questionnaire to assess knowledge, attitudes, and practices related to patient-ventilator asynchrony among ICU health care professionals, with evidence of face and content validity, internal consistency, and test-retest reliability.

## INTRODUCTION

Mechanical ventilation is one of the main reasons for admitting patients to the ICU, aiming to ensure gas exchange, reduce respiratory effort, and improve patient comfort^1^ ^,^ ^2^ while minimizing the risk of further lung injury.^3^ ^-^ ^7^ An adequate interaction between the patient and the mechanical ventilator must therefore be guaranteed, avoiding asynchronies. Patient-ventilator asynchrony (PVA) occurs when there is a mismatch between the patient’s efforts and the ventilator delivery.^8^ ^-^ ^10^

Previous studies have suggested that all patients under mechanical ventilation experience PVA during some period of the ventilatory support,^11^ ^-^ ^15^ and up to 85% of patients may experience PVA in more than 10% of respiratory cycles (high incidence).^16^ ^,^ ^17^ The high incidence of PVA has been associated with increased use of sedatives and neuromuscular blocking agents, as well as with ventilator weaning failure, prolonged mechanical ventilation, prolonged ICU and hospital length of stay, tracheostomy, and potentially mortality.^12^ ^,^ ^15^ ^,^ ^16^ ^,^ ^18^ ^,^ ^19^

ICU health care professionals caring for patients under mechanical ventilation should be aware of clinical signs and ventilation waveform patterns that may reflect the occurrence of PVA. Underrecognition of PVA can prevent proper ventilatory support and cause harm to patients.^3^ ^,^ ^7^ ^,^ ^11^ ^,^ ^20^ However, PVA identification is challenging and requires specific training. In a study evaluating the ability to identify PVA using ventilator waveforms among physicians and residents working in the ICU,^21^ the authors found a low rate of PVA recognition and suggested no association with duration of clinical experience. In addition, it is not well known whether ICU health care professionals do not have sufficient knowledge about PVA (i.e., a knowledge gap), do not believe PVA affects clinical outcomes and warrants routine assessment (i.e., an attitude gap), or do not assess or treat PVA in clinical practice (i.e., a practice gap). 

We sought to create a valid and clinically useful tool to assess ICU health care professionals’ knowledge, attitudes, and practices regarding PVA. Here, we describe the process of development and validation of the questionnaire, as well as its psychometric properties, including item generation based on expert input and literature review; face and content validity assessment; pilot testing and test-retest reliability testing; and adaptations before administration. 

## METHODS

This was a knowledge, attitudes, and practices questionnaire development study. The study protocol was approved by the Research Ethics Committee of the *Instituto do Coração do Hospital das Clínicas da Faculdade de Medicina da Universidade de São Paulo* (Protocol no. 1.886.307), located in the city of São Paulo, Brazil. Online informed consent provided participants with detailed information on the purpose and procedures of the study, as well as on potential risks and benefits, together with confidentiality measures and participant rights. No personal information identifying participants was collected in the questionnaire. Participants could withdraw from the study at any time without consequence. 

The target population for our study was ICU health care professionals, specifically physicians, nurses, respiratory therapists, and physiotherapists involved in the care of mechanically ventilated patients. The inclusion criterion was a clinical workload of ≥ 20 hours per week in the ICU. The only exclusion criterion was declining to provide informed consent. 

For the pilot and test-retest phases of questionnaire validation, we used a convenience sample of eligible ICU professionals working in a medical ICU at the *Instituto do Coração*. The invitation to participate in the study was emailed to all physicians, physiotherapists, and nurses who met the selection criteria. 

Because no validated instrument was available to assess knowledge, attitudes, and practices related to PVA among ICU health care professionals, we developed a questionnaire following a structured, multiphase approach. The questionnaire was developed between 2018 and 2023, a period that included substantial delays due to the COVID-19 pandemic. 

In phase I (conceptual framework and item generation), the first step was the construction of a conceptual framework informed by current literature to guide item generation. Relevant peer-reviewed articles, clinical guidelines, and key concepts in mechanical ventilation and PVA were reviewed to identify the major domains that should be addressed in order to assess PVA knowledge. On the basis of the conceptual framework, an initial pool of items was drafted in simple and neutral language to ensure clarity and relevance across health care disciplines and settings. 

Attitude and practice items were also generated on the basis of current literature and were aligned with the conceptual framework. The attitude items were aimed at assessing respondents’ perceptions of PVA, individually and within their clinical work environment. Practice items were designed to capture real-world behaviors and routines related to the recognition and management of PVA, including how professionals assess PVA, the timing and frequency of evaluations, methods used, interventions applied, and perceived barriers to effective management. In addition, five clinical cases featuring ventilator waveform interpretation were included in a separate section to assess respondents’ ability to apply their knowledge in realistic clinical scenarios. The definitions of PVAs used are described in the supplementary material (Table S1). 

In phase II (face and content validity and reliability), the first draft of the questionnaire was circulated among a panel of five experts in mechanical ventilation (four physicians and one respiratory physiotherapist), who were asked to evaluate each item for clarity, relevance, and representativeness of the intended constructs. Their feedback was synthesized and used in order to revise item wording, eliminate redundant or ambiguous questions, and improve the overall structure and flow of the questionnaire. 

Subsequent revisions were made with the support of an expert in questionnaire development, leading to a series of refinements across multiple versions. By the ninth version, consensus had been reached on the overall structure and content, at which point the questionnaire was reviewed again by a second expert panel, comprising one physician, one respiratory physiotherapist, and one ICU nurse, to ensure multidisciplinary relevance and practical applicability. 

The tenth version of the questionnaire was piloted in Portuguese among a convenience sample of ICU health care professionals to assess the feasibility and reliability of the questionnaire. We invited potential participants using their institutional emails, and only participants who completed at least 80% of the items in both rounds (test and retest) were included in the analysis. To assess test-retest reliability, participants were invited to complete the same questionnaire again 30 days after the initial round. The 30-day interval was chosen to reduce recall effects while minimizing the risk of true changes in knowledge or attitudes over time. Participants completed the questionnaire using a research electronic data capture (REDCap; Vanderbilt University, Nashville, TN, USA) platform.^22^ A demographic section with questions on age, sex, profession, highest level of education, and ICU work experience was also added. 

In phase III (final revision and cross-cultural adaptation) we performed a final revision of the questionnaire based on the pilot test results. The final version of the questionnaire was translated from Portuguese into English and Spanish by using a standardized process: translation by professional translators, followed by independent back-translation into the original language by bilingual experts and review by a panel of experts to ensure semantic, conceptual, and cultural equivalence. Although pretesting with the target population was not conducted, the translated versions underwent comprehensive expert review to enhance clarity and relevance across languages. 

In the final version of the questionnaire, knowledge items were constructed as multiple-choice questions, with four options and only one correct answer. One point was attributed to each correct answer, and zero points were awarded for wrong answers. Attitude items were constructed as statements about PVA, and participants were asked to report on the level of importance, difficulty, or seriousness of the statements using a slider scale going from not very important, difficult, or serious to very important, difficult, or serious. The statements alternate between positive and negative wording to avoid response set bias. Positively worded items were scored from 100 to 0 points, where 100 was attributed to very important/difficult/serious, and zero was attributed to not very important/difficult/serious. We used reverse scores for negatively worded items. For descriptive purposes, the slider scale position was categorized as follows: 0-25, not very important/difficult/serious; 26-50, moderately important/difficult/serious; 51-75, important/difficult/serious; and 76-100, very important/difficult/serious. Practice questions were evaluated qualitatively and were not scored, because they are intended to assess current clinical behaviors and do not have clearly defined correct or optimal responses. For descriptive purposes, practice items with a slider scale were displayed in four categories: 0-25%, 26-50%, 51-75%, and 76-100%. 

A standardized knowledge score was calculated as the sum of the points on the knowledge items divided by the total number of knowledge items and multiplied by 100, resulting in a score ranging from zero to 100. In addition, a standardized attitude score was calculated as the sum of the points on the attitude items divided by the total number of attitude items, resulting in a score ranging from zero to 100. A total score was calculated as the sum of standardized knowledge and attitude scores divided by 2, resulting in a score ranging from zero to 100. 

Descriptive statistics were used in order to summarize participants’ characteristics, questionnaire completion rates, and scores. Continuous variables were presented as median and interquartile range, and categorical variables were presented as percentages. Internal consistency of the knowledge section was assessed with Cronbach’s alpha. Cronbach’s alpha was not calculated for the practice and attitude sections because those items are heterogeneous in format and are not intended to measure a single underlying construct. Test-retest reliability of the knowledge section was evaluated by calculating the intraclass correlation coefficient (ICC). Additionally, Pearson’s correlation coefficient (r) was computed in order to assess the linear association between total scores at test and retest. Comparisons between test and retest were conducted by using a paired t-test or the Wilcoxon signed-rank test for continuous variables, depending on data distribution, as assessed by the Shapiro-Wilk test. McNemar’s test or the Stuart-Maxwell test was performed for paired categorical data. A significance level of p < 0.05 was used. Data processing and analyses were conducted with R software, version 4.4.2 (The R Project for Statistical Computing, Vienna, Austria). 

## RESULTS

The initial version of the questionnaire comprised 69 items, covering PVA knowledge, attitudes, and practices. Following validation and reliability assessment, the final version of the questionnaire comprised 24 items: 14 knowledge items, four attitude items, and six practice items. The knowledge section was designed on the basis of a conceptual framework with six major domains, as described in Chart 1. 

**Chart 1:** Conceptual framework-patient-ventilator asynchrony knowledge.

Domain	Description	Items on the final version of the questionnaire
1. Recognition	ability to interpret real-time ventilator waveforms and identify patterns of PVA	K1, K6, K9, K11, K12
2. Classification	knowledge of common terminology and accurate labeling of PVA types	K2, K7, K10,
3. Mechanisms and Clinical Context	understanding of physiological mechanisms and clinical contributors to PVA	K3, K4, K8
4. Epidemiology	understanding of the prevalence of PVA in clinical practice	K4, K14
5. Clinical Implications	understanding of the potential adverse outcomes related to PVA	K5, K8
6. Diagnostic Approaches	awareness of the most effective methods to detect PVA	K8, K13

PVA: patient-ventilator asynchrony; and K: knowledge item.

A summary of all revisions across versions is presented in Table S2. The first and final multiprofessional panels of experts in mechanical ventilation and critical care agreed that the questionnaire appropriately assessed knowledge, attitudes, and practices related to PVA. Their primary recommendation was to improve clarity through rewording of items. Most of the substantive modifications were guided by the expert in questionnaire development and focused on enhancing structure, consistency, and usability. Key changes included reformatting items for alignment with response types (e.g., multiple choice or Likert scale), eliminating redundant or overly complex items, and incorporating recognition scenarios into the knowledge section to assess applied understanding through ventilator waveform interpretation. 

The tenth version of the questionnaire consisted of 15 knowledge items, four attitude items, and six practice items. Chart 2 presents the specific component targeted by each item. Of the 83 ICU health care professionals who consented to participate, 62 (75%) completed at least 80% of the questionnaire in the first round (test). Forty-nine respondents (59%) completed at least 80% of the second round (retest) 30 days after the initial testing and were included in the reliability analysis. Of those, 29 (59%) were female, 16 (33%) were physicians, 10 (20%) were nurses, and 23 (47%) were respiratory therapists/physiotherapists. The median ICU experience was 4 [2-8] years (Figure S1). 

**Chart 2:** Specific domains targeted by each questionnaire item.

Section	Item	Key component
Knowledge	K1	Recognition of ineffective effort
Knowledge	K2	Classification of ineffective effort
Knowledge	K3	Identification of potential causes of PVA
Knowledge	K4	Understanding when PVA can occur
Knowledge	K5	Awareness of clinical outcomes associated with PVA
Knowledge	K6	Recognition of flow asynchrony
Knowledge	K7	Classification of flow asynchrony
Knowledge	K8	Identification of clinical signs associated with PVA
Knowledge	K9	Recognition of cycling asynchrony
Knowledge	K10	Classification of cycling asynchrony
Knowledge	K11	Recognition of normal ventilator waveform patterns
Knowledge	K12	Recognition of double triggering
Knowledge	K13	Selection of appropriate methods to assess PVA
Knowledge	K14	Knowledge of the estimated prevalence of PVA in a medical ICU
Knowledge	K15*	Knowledge of the estimated prevalence of PVA in a surgical ICU
Attitudes	A1	Importance of assessing patient-ventilator interaction
Attitudes	A2	Importance of observing ventilator waveforms
Attitudes	A3	Severity of asynchrony in the ICU
Attitudes	A4	Difficulty interpreting ventilator waveforms
Practices	P1	Frequency of routine assessment of patient-ventilator interaction
Practices	P2	Duration of waveform observation during assessment
Practices	P3	Timing of assessment during mechanical ventilation
Practices	P4	Frequency of ventilator adjustments in response to detected PVA
Practices	P5	Frequency of sedation adjustments in response to detected PVA
Practices	P6	Perceived barriers to assessing and managing PVA

PVA: patient-ventilator asynchrony. *this item was removed after the test-retest phase, in order to improve internal consistency, with expert feedback.

**Figure 1:**
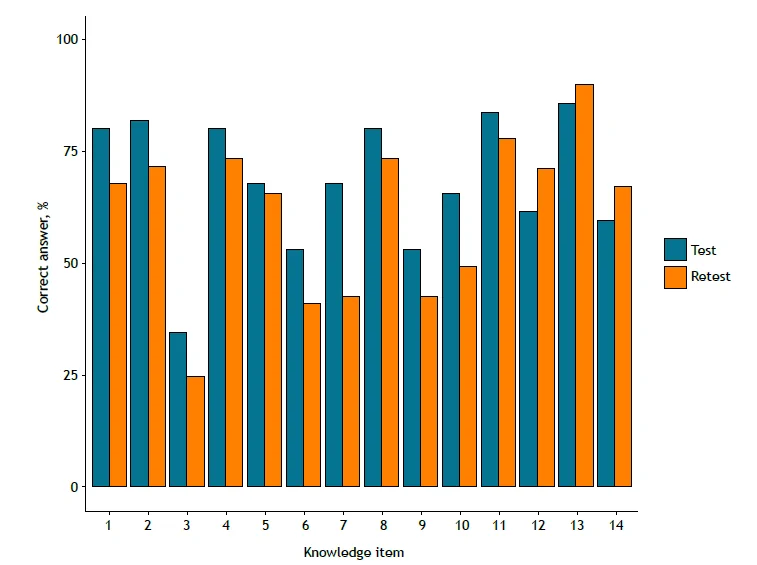
Proportion of correct answers for each knowledge item.

The knowledge section showed acceptable internal consistency, with a Cronbach’s alpha of 0.73. Reliability improved slightly to 0.74 after removal of item K15. On the basis of that improvement and expert feedback, item K15 was removed from the final version in order to enhance internal consistency. After removing K15, the ICC for the total knowledge score was 0.48 (95% CI, 0.24-0.67; p < 0.001), indicating moderate test-retest reliability and acceptable agreement between rounds. The median knowledge score was 71 [57-79] at test and 64 [50-79] at retest (p = 0.052). A moderate, statistically significant positive correlation was observed between test and retest knowledge scores (r = 0.51; 95% CI, 0.27-0.69; p < 0.001), suggesting reasonable consistency across rounds. Figure 1 displays the proportion of correct answers for each knowledge item. 

**Figure 2:**
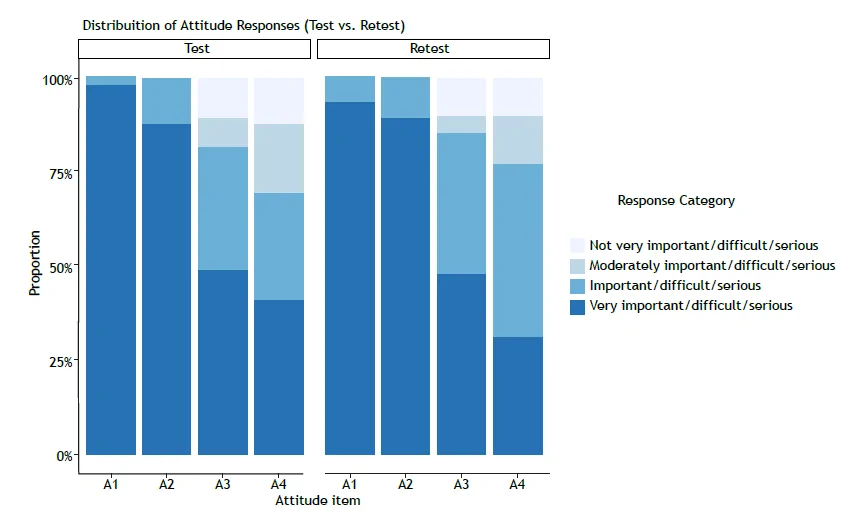
Descriptive analysis of attitude items at test and retest.

The median attitude score was 75 [69-80] at test and 75 [68-79] at retest (p = 0.639). The Spearman correlation between test and retest attitude scores was 0.60 (p < 0.001). The ICC for the total attitude score was 0.68 (95% CI, 0.50-0.81; p < 0.001), indicating good test-retest reliability. Figure 2 describes the responses to each attitude item. 

Practice item responses for the test and retest are presented in Table 1. For item P6, participants had the opportunity to describe “other” barriers to managing PVA through an open-ended response option. Although no consistent new theme emerged across responses to justify the creation of an additional category, participants highlighted critical individual challenges, such as the absence of routine assessment, limitations in ventilator capabilities, reluctance to adjust sedation practices, and issues related to interprofessional collaboration. 

**Table 1:** Descriptive analysis of practice items at test and retest.^a^

Practice item	Test (n = 49)	Retest (n = 48)	p
P1. Frequency of routine assessment of patient-ventilator interaction			0.172
0-25%	4 (8%)	1 (2%)	
26-50%	5 (10%)	5 (10%)	
51-75%	10 (20%)	11 (23%)	
76-100%	30 (61%)	31 (65%)	
P2. Duration of waveform observation during assessment			0.194
For less than 10 s (less than 3 breaths)	1 (2%)	3 (6%)	
For about 10 s or 3 or 4 breaths	11 (22%)	6 (13%)	
For about 15 s or 6 or 8 breaths	16 (33%)	21 (44%)	
For about 30 s or 10 breaths	21 (43%)	18 (38%)	
P3. Timing of assessment during mechanical ventilation			0.572
Only during controlled ventilation	1 (2%)	3 (6%)	
Only during assisted and/or spontaneous ventilation	3 (6%)	3 (6%)	
Only during weaning from mechanical ventilation	0	1 (2%)	
During the entire period of mechanical ventilation	45 (92%)	41 (92%)	
P4. Frequency of ventilator adjustments in response to detected PVA			0.123
0-25%	1 (2%)	1 (2%)	
26-50%	5 (10%)	0	
51-75%	4 (8%)	8 (17%)	
76-100%	39 (80%)	39 (81%)	
P5. Frequency of sedation adjustments in response to detected PVA			0.349
0-25%	12 (25%)	9 (19%)	
26-50%	12 (25%)	8 (17%)	
51-75%	14 (28%)	14 (29%)	
76-100%	11 (22%)	17 (35%)	
P6. Perceived barriers to assessing and managing PVA			
Lack of time	12 (25%)	11 (22%)	> 0.999
Lack of knowledge of who is responsible	25 (51%)	24 (49%)	> 0.999
Impression that PVA is not a cause of worse outcomes	10 (20%)	10 (20%)	> 0.999
Other	14 (29%)	8 (16%)	0.181

PVA: patient-ventilator asynchrony. ^a^Data presented as n (%).

The final version of the questionnaire consisted of 24 items, including 14 knowledge items, four attitude items, and five practice items. The finalized questionnaire was then translated into Spanish and English. All three language versions are available in the supplementary material. 

## DISCUSSION

We developed a questionnaire to assess knowledge, attitudes, and practices regarding PVA among ICU health care professionals. The final version included 14 knowledge items, four attitude items, and five practice items, covering essential domains such as recognition, classification, clinical implications, and management strategies for PVA. 

The questionnaire was constructed on the basis of literature review and expert input from mechanical ventilation specialists and an expert in questionnaire development. Panels confirmed that the instrument adequately captured key aspects of PVA, and revisions primarily focused on improving clarity, reducing redundancy, and ensuring feasibility. Those refinements resulted in a practical tool suitable for clinical settings. This is supported by our finding that 75% of respondents completed at least 80% of the questionnaire during the initial round, suggesting acceptable usability.^23^ ^,^ ^24^

The knowledge section showed acceptable internal consistency (Cronbach’s alpha = 0.73), which is reasonable given the multidimensional nature of PVA.^25^ ^,^ ^26^ While instruments focused on a single domain may achieve higher reliability, our broader approach enhances clinical relevance and utility. To address this complexity, we provided a detailed mapping of each item to its conceptual component, allowing future users to conduct more targeted assessments if needed. Test-retest reliability ranged from moderate to good, indicating stability over time.^27^ ^,^ ^28^ These findings align with previous literature emphasizing the challenges of achieving high reliability in multidomain clinical questionnaires.^29^ ^,^ ^30^ The practice domain was designed to descriptively assess current clinical behavior and can be interpreted as a monitoring tool to characterize patterns and identify potential areas for improvement. 

Our questionnaire offers a foundation for identifying gaps in PVA-related knowledge, attitudes, and practices across different professional groups and care settings. Such information is essential to design tailored educational programs, implement cultural and behavioral interventions, and evaluate the impact of practice changes or quality improvement initiatives. The inclusion of attitude and practice sections enhances the tool’s value beyond knowledge assessment,^31^ ^,^ ^32^ supporting a more comprehensive understanding of barriers and facilitators to optimal PVA management. 

The questionnaire was translated into Spanish and English, enhancing its global applicability. While pretesting of these versions was not performed, all translations underwent extensive review by bilingual health care professionals familiar with mechanical ventilation. This step supports linguistic and conceptual equivalence, although additional validation in diverse settings is warranted. Our next goal is to conduct multicenter, international studies to further validate the instrument and enable its widespread use. 

To the best of our knowledge, this is the first instrument developed with evidence of validity to simultaneously assess knowledge, attitudes, and practices related to PVA among ICU health care professionals. The lack of a validated tool to date has hindered objective measurement of these competencies, and our questionnaire addresses this critical gap in the literature. 

The present study has several limitations. First, it was conducted in a single center, which may limit the generalizability of the results. Because of the characteristics of the health care team, the ICU protocols, and the institutional culture of the center, they may not be representative of other hospitals, either nationally or internationally. Second, the sample used for the pilot study was a convenience sample, and there was a noticeable loss to follow-up between test and retest, which may have introduced bias and influenced reliability. It is possible that the ICU health care professionals who volunteered to participate and completed the test and retest were more interested in or knowledgeable about mechanical ventilation and PVA, which could lead to an overestimation of the knowledge levels and attitudes of the general population of ICU health care professionals. Third, we did not perform advanced psychometric analyses such as exploratory factor analysis or calculate formal content validity indices. Although this may limit the quantitative strength of the content validity evidence, the study incorporated multiple methodological steps to support initial instrument development and reliability assessment. Fourth, although translation and expert review were performed, the absence of cognitive debriefing or pretesting with target-language users may limit full confirmation of semantic and contextual equivalence. Additionally, formal usability metrics were not assessed. Finally, the field of mechanical ventilation and the understanding of PVA are constantly evolving. Consequently, the questionnaire’s content may require periodic updates to remain relevant and aligned with new evidence and clinical guidelines. 

In conclusion, we developed a comprehensive and user-friendly questionnaire to assess knowledge, attitudes, and practices related to PVA among ICU health care professionals, with evidence of face and content validity, internal consistency, and test-retest reliability. In the pilot sample, we found that median knowledge and attitude scores exceeded 70 out of 100, suggesting satisfactory baseline understanding and perception of PVA, and reported barriers to managing PVA included issues related to interprofessional collaboration. The questionnaire can be used in large-scale, international studies to describe knowledge, attitudes, and practices regarding PVA. 
